# Covalent Inhibition of Pyruvate Kinase M2 Reprograms Metabolic and Inflammatory Pathways in Hepatic Macrophages against Non-alcoholic Fatty Liver Disease

**DOI:** 10.7150/ijbs.73890

**Published:** 2022-08-15

**Authors:** Ni Fan, Xiuying Zhang, Wei Zhao, Jia Zhao, Dan Luo, Yilu Sun, Ding Li, Chenliang Zhao, Yu Wang, Hongjie Zhang, Jianhui Rong

**Affiliations:** 1 School of Chinese Medicine, Li Ka Shing Faculty of Medicine, The University of Hong Kong, Hong Kong, China.; 2 College of Chemistry and Pharmacy, Northwest A&F University, Shaanxi, China.; 3 School of Chinese Medicine, Hong Kong Baptist University, Kowloon Tong, Hong Kong, China.; 4 Department of Pharmacology and Pharmacy, Li Ka Shing Faculty of Medicine, The University of Hong Kong, Hong Kong, China.; 5 The University of Hong Kong Shenzhen Institute of Research and Innovation (HKU-SIRI), Shenzhen, China.

**Keywords:** macrophage polarization, pyruvate kinase M2, celastrol, covalent modification, non-alcoholic fatty liver disease

## Abstract

Warburg effect of aerobic glycolysis in hepatic M1 macrophages is a major cause for metabolic dysfunction and inflammatory stress in non-alcoholic fatty liver disease (NAFLD). Plant-derived triterpene celastrol markedly inhibited macrophage M1 polarization and adipocyte hypertrophy in obesity. The present study was designed to identify the celastrol-bound proteins which reprogrammed metabolic and inflammatory pathways in M1 macrophages. Pyruvate kinase M2 (PKM2) was determined to be a major celastrol-bound protein. Peptide mapping revealed that celastrol bound to the residue Cys^31^ while covalent conjugation altered the spatial conformation and inhibited the enzyme activity of PKM2. Mechanistic studies showed that celastrol reduced the expression of glycolytic enzymes (e.g., GLUT1, HK2, LDHA, PKM2) and related signaling proteins (e.g., Akt, HIF-1α, mTOR), shifted aerobic glycolysis to mitochondrial oxidative phosphorylation and skewed macrophage polarization from inflammatory M1 type to anti-inflammatory M2 type. Animal experiments indicated that celastrol promoted weight loss, reduced serum cholesterol level, lipid accumulation and hepatic fibrosis in the mouse model of NAFLD. Collectively, the present study demonstrated that celastrol might alleviate lipid accumulation, inflammation and fibrosis in the liver via covalent modification of PKM2.

## Introduction

Chronic inflammation and metabolic disorders hallmark and drive the pathogenesis and progression of non-alcoholic fatty liver disease (NAFLD) to non-alcoholic steatohepatitis (NASH), fibrosis, irreversible cirrhosis, and even hepatocellular carcinoma 1-3. Dyslipidemia initiates the onset of NAFLD and exacerbates the inflammation, damage and ballooning degeneration of hepatocytes 3, 4. Upon the lipotoxic stimulation, inflammatory macrophages are activated and mediate the inflammatory response and hepatic injury 5. Macrophages display phenotypic and functional plasticity and undergo inflammatory M1 polarization or anti-inflammatory M2 polarization under specific pathologic conditions 6. Inflammatory M1 macrophages are the predominant phenotype in NAFLD/NASH, secrete several proinflammatory cytokines (e.g., IL-1β, IL-6, and TNF-α) and promote the progression of hepatic steatosis to NASH, fibrosis and cirrhosis. Interestingly, M2 macrophages release anti-inflammatory cytokines (e.g., IL-4, IL-10), attenuate hepatic injury and promote tissue repair 7. Timely switch of macrophage polarization from M1 to M2 phenotype may determine the resolution of hepatic inflammation in NAFLD/NASH.

The phenotypes and functions of macrophages are tightly associated to the metabolic status 8, 9. The metabolism of M1 macrophages is signatured by the hyperactivation of fatty acid synthesis, glycolysis**,** lipid uptake and pentose phosphate pathway, and the suppression of tricarboxylic acid (TCA) cycle and mitochondrial respiration 10. By contrast, the metabolism of M2 macrophages is characterized by fatty acid oxidation and mitochondrial respiration 11, 12. Glycolysis is tightly regulated for generating ATP and biosynthetic intermediates**.** Pyruvate kinase (PK) is one of the regulatory enzymes in glycolysis and catalyzes the phosphate transfer from phosphoenolpyruvate to ADP to yield pyruvate and ATP 13. Unlike other isoforms, PKM2 plays a key role in the metabolic reprogramming of cancer cells and activated immune cells 14, 15. On the other hand, PKM2 may be phosphorylated and translocated to the cell nucleus, and thereby regulate the transcriptional activity of hypoxia-inducible factor 1α (HIF1α) in the induction of inflammatory factors and glycolytic enzymes (e.g., GLUT1, LDHA) 15, 16. Pharmacological or genetic inhibition of PKM2 could effectively ameliorate inflammation via reducing the production of lactate, the release of proinflammatory cytokines, and the formation of inflammasome 16. Importantly, it was recently demonstrated that PKM2 was greatly expressed in the livers, especially in hepatic macrophages of NAFLD and NASH patients 17. Thus, PKM2 may be a promising target for therapeutic intervention in NAFLD and NASH.

Pharmacological approaches have been evaluated for skewing macrophage polarization from M1 to M2 phenotype 18. Natural product pentacyclic triterpene celastrol exhibits a spectrum of pharmacological activities against chronic diseases and demonstrates the best potency against obesity 19, 20. Importantly, celastrol appears to be effective for treating metabolic disorders in obesity, diabetes, NAFLD and NASH 21. Several previous studies suggest that celastrol may target different molecular mechanisms as follows: 1) to increase leptin sensitivity by reducing endoplasmic reticulum (ER) stress 22 and inactivating the NF-kB pathway 23; 2) to promote energy expenditure and mitochondrial function by activating HSF1-PGC1α transcriptional axis 24; 3) to alleviate inflammation by suppressing Nur77 mediated autophagy 25; 4) to promote weight loss via improving antioxidant capacity and lipid metabolism 26, 27. Along this line, we previously reported that celastrol might ameliorate inflammation in obese mice via reducing macrophage M1 polarization 28. Presumably, celastrol targets different signaling pathways via interacting with the relevant protein targets 21, 29. On the other hand, celastrol possesses the quinone methide moiety with the possibility to covalently modify various protein targets. The recently emerged chemical biology approaches may support the identification of celastrol-bound proteins as the therapeutic targets for developing anti-NAFLD drugs.

In the present study, celastrol-PEG3-azide was synthesized and immobilized onto alkyne-coated agarose resin under click chemistry condition for the pulldown of celastrol-bounds proteins. We further investigated the role of the celastrol-bound proteins in reprogramming macrophage polarization in the mouse model of NAFLD/NASH.

## Materials and Methods

### Chemicals and antibodies

Celastrol was supplied by Nanjing Spring & Autumn Biological Engineering Company (Nanjing, Jiangsu, China). Antibodies against PKM2, GLUT1, LDHA, phospho-mTOR, mTOR, phospho-AKT, AKT, COX-2 and anti-mouse HRP-conjugated IgG secondary antibody were obtained from Cell Signaling Technology (Danvers, MA, USA). HK-2 antibody was purchased from Abclonal (Cambridge, MA, USA). Antibodies against Arginase-1 and protein A/G PLUS-agarose were purchased from Santa Cruz Biotechnology (Dallas, TX, USA). Antibodies against IL-1β, beta-actin, HIF1α, PNCA, iNOS and HRP-conjugated streptavidin were acquired from Thermo Fisher Scientific (Waltham, MA, USA). FITC‐conjugated anti‐mouse CD86 antibody and APC-conjugated anti-mouse CD206 antibody were purchased from eBioscience (San Diego, CA, USA). ECL™ Detection Reagents, RIPA buffer and chemicals otherwise indicated were obtained from Sigma-Aldrich (St. Louis, MO, USA). For details of all reagents, please refer to Table S1 in the supplementary file.

### Cell culture and drug treatment

Murine macrophage cell line RAW264.7 was purchased from American Type Culture Collection (Manassas, VA, USA). The macrophages were maintained in DMEM medium containing 10% FBS and 1% penicillin/streptomycin at 37 °C in a humidified incubator containing 5% CO_2_. For drug treatment, 2x10^5^ cells/ml of cells were seeded in 6-well plate for 24 hours and treated with celastrol at the various concentrations for 1 hour followed by stimulating with LPS (1 μg/mL) for another 24 hours.

### Chemical synthesis of celastrol-PEG3-azide

Celastrol-PEG3-azide was synthesized by coupling celastrol with azide-PEG3-amine and purifying with silica gel chromatographic column as previously described 30. ^1^H NMR (400 MHz, chloroform-*d*) *δ* 7.01 (d, *J* = 7.0 Hz, 1H), 6.49 (s, 1H), 6.37 (t, 1H), 6.33 (d, *J* = 7.0 Hz, 1H), 3.66 - 3.63 (m, 4H), 3.61- 3.59 (m, 2H), 3.57 - 3.52 (m, 2H), 3.48 - 3.43 (m, 2H), 3.43 - 3.38 (m, 2H), 3.32 - 3.29 (m, 2H), 2.19 (s, 3H), 2.15 - 1.93 (m, 4H), 1.91- 1.79 (m, 3H), 1.67- 1.60 (m, 3H), 1.59 - 1.52 (m, 3H), 1.45 - 1.51 (m, 2H), 1.41 (s, 3H), 1.24 (s, 3H), 1.13 (s, 3H), 1.10 (s, 3H), 1.05 - 0.96(m, 2H), 0.60 (s, 3H). ^13^C NMR (101 MHz, CDCl_3_) *δ* 178.38, 178.37, 170.72, 165.00, 146.09, 134.45, 127.40, 119.53, 118.15, 117.37, 70.65, 70.53, 70.47, 70.08, 70.03, 69.98, 50.76, 45.18, 44.46, 43.15, 40.41, 39.45, 39.28, 38.30, 36.46, 35.02, 33.85, 33.63, 31.72, 31.16, 30.89, 30.11, 29.47, 28.76, 21.81, 18.41, 10.38. HRMS (m/z): [M+ H]^ +^ calcd. for C_37_H_55_N_4_O_6_, 651.4122; found 651.4005.

### Identification of celastrol-bound proteins

**Figure 1:**
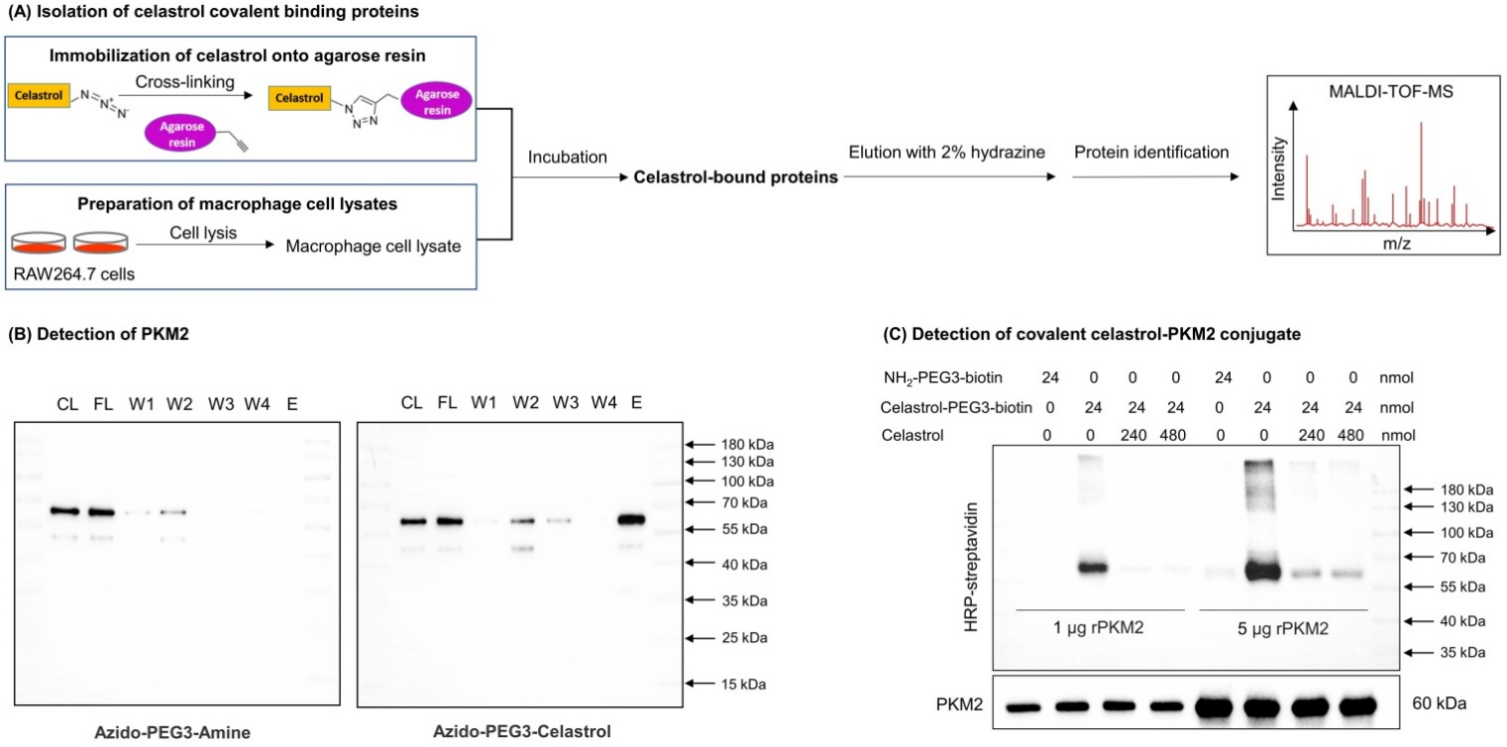
** Identification of PKM2 as a major celastrol-modified protein. (A)** Scheme for the isolation of covalent celastrol-protein conjugates. After the incubation of macrophage proteins with celastrol-coated agarose resin, celastrol-bound proteins were eluted, separated and characterized by MALDI-TOF-MS technology. **(B)** WB analysis of celastrol-bound proteins. The proteins were detected by WB with anti-PKM2 antibody. CL: cell lysates; FL: flow-through fraction; W1-W4: washing fractions; E: elution fraction. **(C)** Validation of covalent celastrol-PKM2 conjugate. Recombinant PKM2 was in-house prepared and treated with celastrol-PEG3-biotin with or without celastrol at 4 °C overnight. The celastrol-PKM2 conjugate were examined by WB analysis using streptavidin-HRP or anti-PKM2 antibody.

Covalent celastrol-protein conjugates were isolated and identified as outlined in Fig. 1A using the Click-&-Go Dde Protein Enrichment Kit (Click Chemistry Tools). Briefly, the alkyne agarose resin was coated with azide-PEG3-amine or celastrol-PEG3-azide under click chemistry reaction conditions with copper catalyst. For the preparation of the cellular proteins, RAW264.7 cells (total 10 dishes) were stimulated with LPS and subsequently lysed with RIPA buffer containing proteinase inhibitors. After vigorous centrifugation, the supernatant was collected and desalted by passing through PD-10 column from GE Healthcare. The cellular proteins were incubated with azide-PEG3-amine- or celastrol-PEG3-azide-coated agarose resin at 4 °C overnight, separated by centrifugation, washed 4 times with washing solution and eluted with 2% hydrazine. The protein fractions were resolved by electrophoresis on 10% SDS polyacrylamide gel and stained with Coomassie Brilliant Blue R-250 or immunoblotted with antibodies against PKM1 or PKM2. The celastrol-bound proteins were identified as previously described 30.

### Preparation of recombinant PKM1, PKM2 and mutated PKM2 (C31S) protein

The full length cDNAs encoding PKM1 and PKM2 were cloned from pLHCX-Flag-mPKM1 (Plasmid #42511, Addgene) and pLHCX-Flag-mPKM2 (Plasmid #42512, Addgene) by PCR technique using forward primer: 5'- GTACGAATTCATGCCGAAGCCACACAGT-3'; reverse primer: 5'- GTACCTCGAGTCAAGGTACAGGCACTACAC-3'. Following cleavage with restriction enzymes XhoI and EcoRI, the PCR products were respectively ligated into bacterial expression vector pET-28a with T4 DNA ligase. For the generation of mutated PKM2 (C31S) plasmid, the point mutation was achieved using Mut Express II Fast Mutagenesis Kit V2 (Nanjing Vazyme Biotech Co., Ltd) with forward primer 5′-GGACATGTGTTCCAGGAAGGTGTCAGCCATGG-3′ and reverse primer 5′-TCCTGGAACACATGTCCCGCCTGGACATTGACTCTG-3′. The construction of expression plasmid was confirmed by DNA sequencing at BGI Hong Kong Company Limited (Hong Kong, China). The cDNA constructs were introduced into *E. coli* BL21 (DE3) cells while the bacterial colonies were examined for transformation. For protein expressions, the best *E. coli* BL21 (DE3) transformants were induced with 0.5 mM isopropyl β-D-1-thiogalactopyranoside (IPTG) at 30 °C for 6 h. The recombinant proteins were purified with a HisTrap^TM^ HP column under the control of ÄKTAexplorer™ chromatography system from GE Healthcare. The recombinant protein fractions were combined and stored at -80 °C until use.

### Validation of covalent celastrol-PKM2 conjugate

Celastrol-PEG3-biotin was prepared and characterized as previously described 30. For detecting the binding of celastrol to PKM2 or mutated PKM2 (C31S), the recombinant proteins in HEPES buffer were treated with excessive celastrol-PEG3-biotin or celastrol at 4 °C for overnight and then detected by western blot (WB) analysis. The covalent celastrol-PKM2 conjugate was detected with HRP-Conjugated streptavidin and enhanced chemiluminescence (ECL) reagent, while the protein input was assessed by WB analysis using anti-PKM2 antibody.

### Proteomic identification of celastrol-bound peptides

Celastrol (30 µg) was incubated with recombinant PKM2 protein (40 µg) in HEPES buffer (10 mM, containing 100 mM KCl, 5 mM MgCl_2_, 1% DMSO, pH 7.8) at 4 °C overnight. The reaction mixture was resolved by SDS-PAGE and stained with Coomassie brilliant blue. The protein band was recovered and subjected to in-gel digestion with trypsin. The peptides were desalted by Ziptip C18 resin and analyzed by LC-MS/MS method as previously described 30.

### Molecular docking

The crystal structure of PKM2 (4B2D) was downloaded from the Protein Data Bank (https://www.rcsb.org). Covalent docking of celastrol to the structure of PKM2 was performed with the software AutoDock 4 as previously described 31. With the two-point attractor method, celastrol was modeled as a free ligand, while aa optimal potential was used to bring together the quinone methide moiety of celastrol and the amino acid residue Cys^31^ in PKM2. The celastrol-PKM2 conjugate was visualized by PyMOL (hppt://www.pymol.org) and Molecular Operating Environment (MOE, https://www.chemcomp.com/Products.htm).

### Assay for the enzymatic activities of PKM1 and PKM2

The recombinant PKM1 or PKM2 was incubated with celastrol at the indicated concentrations. At the end of reaction, the enzymatic activities of PKM1 and PKM2 were assayed using BioVision pyruvate kinase activity colorimetric/fluorometric assay kit and presented as a percentage compared to the vehicle-treated controls. The IC_50_ value was calculated by a sigmoidal dose-response curve on GraphPad Prism software.

### Visualization of the covalent celastrol-PKM2 conjugate in macrophage cells

Celastrol-PEG4-Alkyne was prepared and characterized as previously described 30. For *in vitro* formation of covalent celastrol-PKM2 conjugate, the cells were treated with celastrol-PEG4-Alkyne or celastrol for 2 h. The cellular deposition of celastrol was traced by click chemistry labeling with AFDye555-picolyl azide whereas the cellular PKM2 was visualized by immunofluorescence staining. The intracellular localization of PKM2 and the deposition of celastrol were imaged by Zeiss LSM 800 confocal microscopy (Carl-Zeiss, Jena, Germany).

### Flow cytometry

After drug treatment, RAW264.7 cells were firstly stained with anti-CD86 antibody. For the detection of CD206, the cells were fixed and permeabilizated with Cytofix/Cytoperm™ Fixation/Permeabilization Kit (BD Biosciences). The cells were subsequently stained with anti-CD206 antibody. Flow cytometric analysis was performed on NovoCyte Quanteon Flow Cytometer from Agilent Technologies. Data were analyzed with NovoExpress Software.

### Seahorse assay for glycolytic and mitochondrial functions

Oxygen consumption rate (OCR) and extracellular acidification rate (ECAR) of live cells were examined with Seahorse XF Glycolysis Stress Test Kit and Seahorse XF Cell Mito Stress Test Kit on Agilent Seahorse XFe96 Analyzer. RAW264.7 cells were seeded at the density of 8000 cells per well in XFe96 cell culture microplates. For ECAR analysis, 10 mM glucose, 1.5 μM oligomycin, 100 mM 2-deoxy-glucose (2-DG) were sequentially injected into each well. For OCR measurement, 1.5 μM oligomycin, 2.5 μM FCCP and 2.5 μM rotenone/antimycin A were sequentially injected. The data were analyzed and normalized against the cell counts using the Seahorse XFe Wave software.

### Preparation of cellular/hepatic proteins, cytosolic and nuclear proteins

The cellular/hepatic proteins were extracted using the same method as previous described 30. The cytosolic and nuclear proteins were isolated using commercial extraction reagents. Briefly, the cells were lysed with ice-cold cytoplasmic extraction reagent for 10 min. After centrifugation, the supernatants were recovered as the cytosolic proteins, whereas pellets were suspended in ice-cold nuclear extraction reagent for 40 min and centrifuged to recover the supernatants as the nuclear proteins.

### Western blot (WB) analysis

The cellular proteins (20-30 μg) or hepatic proteins (60 μg) were separated by 8-12% SDS-PAGE and subsequently transferred onto polyvinylidene difluoride (PVDF) membranes. After blocking with 5% BSA in TBST solution at 4 °C overnight, the blots were probed with specific primary antibodies and detected with HRP-conjugated secondary antibody. The blots were detected by ECL detection reagents.

### Immunofluorescence staining

For immunofluorescence staining of RAW264.7 cells, the procedures were performed as previously described 30. For immunofluorescence staining of liver tissues, the livers were collected from the experimental mice, fixed in 4% PFA and dehydrated in 30% sucrose in PBS. After frozen at -20 °C, the liver tissues were cut into the sections at 12 μm thickness. The liver sections were subjected to antigen retrieval, permeabilization and blocking as previously described 30. The tissue slices were then incubated with primary antibodies against F4/80, iNOS, arginase-1 and PKM2 and then incubated with corresponding secondary antibodies. The cell nuclei were detected with DAPI. The cells or the liver sections were imaged on the confocal microscopy.

### Co-immunoprecipitation

After drug treatment, RAW264.7 cells were lysed by RIPA buffer. The PKM2-HIF-1α complex was isolated with PKM2 antibody on protein A/G PLUS-agarose overnight. Non-specific rabbit IgG antibody was used as negative control. The pulldown proteins were examined using WB assay with anti-HIF-1α antibodies. The cell lysates were detected with anti-PKM2 antibodies as the index of protein loading.

### Animal experiments

The animal experiments were performed following the guidelines of the Committee on the Use of Live Animals in Teaching and Research of the University of Hong Kong. The NAFLD model was established by feeding mice with high fat diet (HFD) as previously described 32. In brief, male C57BL/6N mice (3~4 weeks, 11-13 g) were fed with 60 kcal% HFD for consecutively 12 weeks, whereas the control mice were fed on chow diet. For the drug treatment, celastrol was dissolved in saline containing 5% DMSO and 1% Tween-20. Celastrol (3.5 mg/kg/d) was orally administrated to the HFD mice for 21 consecutive days. After drug treatment, the mice were weighed daily. On the final day of drug administration, mice were analyzed for the fat contents and lean contents as previously described 33. The serum samples were collected for the analysis of total cholesterol (TC), triglyceride (TG), aspartate transaminase (AST) and alanine transaminase (ATL) using the commercial diagnostic reagent kits from Stanbio Laboratory.

### Tissue staining by Hematoxylin and eosin (H&E) stain, Oil Red O stain and Picrosirius Red stain

On the final day of drug administration, the tissues were collected and examined by H&E staining, Oil Red O staining and Picrosirius Red staining as previously described 33, 34. In brief, hepatic tissues were fixed in 4% PFA, embedded in paraffin and cut into the sections. Paraffin-embedded sections were dewaxed, rehydrated and stained with H&E stain or Picrosirius Red stain. For Oil Red O staining, the livers were fixed in 4% PFA, dehydrated in 30% sucrose. After frozen at -20 °C, the liver tissues were cut into the sections at 12 μm thickness. The liver sections were incubated in 0.2% Oil Red O reagent for 20 min. The images were acquired on Olympus BX43F light microscope (Tokyo, Japan).

### Quantitative real-time polymerase chain reaction (qRT-PCR)

The hepatic total RNAs were isolated with TRIzol reagent and reversely transcribed to the cDNAs with commercial cDNA synthesis kit. The mRNA levels of inflammatory cytokines were determined by qRT-PCR with the specific primers and SYBR Green mix from QIAGEN on the LightCycler^®^ 480 System from Roche Diagnostics International AG (Rotkreuz, Switzerland). The mRNA levels of different biomarkers were normalized against the mRNA levels of β-actin.

### Statistical analysis

The results were expressed as mean ± SD for all experiments. Statistical analysis was performed using one-way ANOVA followed by Tukey test or two-way ANOVA followed by Sidak test with GraphPad Prism software (La Jolla, CA, USA). A *p*-value less than 0.05 was considered as statistically significant.

## Results

### PKM2 was determined to be predominant celastrol-bound protein

A small molecule probe celastrol-PEG3-azide was synthesized to isolate celastrol-bound proteins through the procedure as outlined in Fig. 1A. Celastrol-PEG3-azide was coupled to alkyne-coated agarose resin with a cleavable linker via click chemistry cycloaddition reaction. The celastrol-coated agarose resin was then incubated with cellular proteins isolated from LPS-stimulated RAW264.7 cells to allow the covalent binding to occur. The covalently bound proteins were released from the agarose resin by hydrazine-mediated cleavage. After separating by gel electrophoresis and visualizing by Coomassie blue dye, the protein bands were recovered, digested by trypsin and identified by MALDI-TOF-MS technology. PKM2 was identified as a predominant celastrol-bound protein from the 55-70 kDa protein band (Fig. S1). As shown in Fig. 1B and Fig. S2A, WB analysis with specific antibodies showed an evident signal for PKM2 while no signal for PKM1 in the same protein band. These results suggested that celastrol preferentially formed covalent conjugate with PKM2 rather than PKM1.

### Celastrol covalently modified PKM2 and diminished the enzyme activity

**Figure 2:**
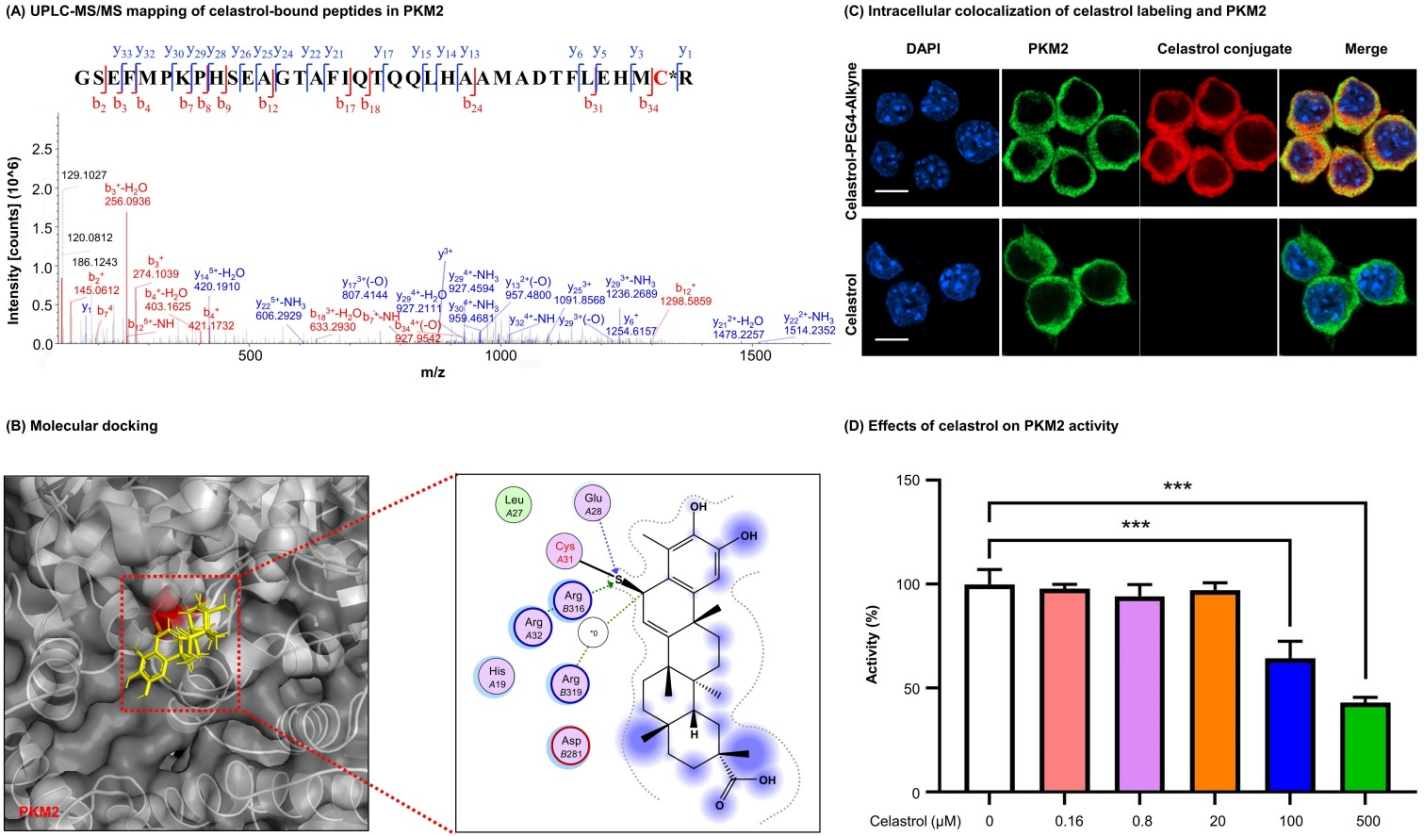
** Characterization of covalent celastrol-binding site in PKM2 and the impact on pyruvate kinase activity. (A)** UPLC-MS/MS peptide mapping of covalent celastrol binding peptides from recombinant PKM2. Following overnight incubation with celastrol, recombinant PKM2 solution was resolved with SDS-PAGE, stained with Coomassie blue and characterized by UPLC-MS/MS method. **(B)** *In silico* prediction of molecular docking. **(C)** Formation and visualization of intracellular covalent celastrol-PKM2 conjugate. After incubating RAW264.7 cells with celastrol-PEG4-alkyne or celastrol, the conjugated celastrol-PEG4-alkyne was visualized with AFDye555-picolyl azide while the cellular PKM2 was visualized by immunofluorescence staining. The cell nuclei were detected with DAPI. Scale bar: 10 µm. **(D)** Inhibition of PKM2 activity by celastrol. After overnight incubation with celastrol, recombinant PKM2 protein was assayed for pyruvate kinase activity. ***p < 0.001 (celastrol vs control).

To visualize the covalent binding of celastrol to PKM2 with the biotin-streptavidin detection system, celastrol-PEG3-biotin was synthesized as a small molecule probe while the biotin tag was detected by WB analysis with HRP-streptavidin. As a result, celastrol-PEG3-biotin was readily conjugated to PKM2 and detected by WB analysis (Fig. 1C). However, such biotin detection was fully prevented by a ten-fold excess of free celastrol, supporting the stable covalent linkage between celastrol and PKM2 by nature. To identify the amino acid residues for covalent attachment by celastrol, we incubated celastrol with recombinant PKM2 and analyzed the protein complex by LC-MS/MS technique. The peptide with the sequence of GSEFMPKPHSEAGTAFIQTQQLHAAMADTFLEHMC^31^R was modified by celastrol (Fig. 2A). Specifically, the Cys^31^-containing fragments from y3 to y33 were accordingly increased by the mass of 450.61 Da for celastrol, suggesting the residue Cys^31^ as the attachment site for celastrol. Subsequently, we performed covalent docking using the AutoDock Tools to simulate the binding mode between celastrol and PKM2 (PDB ID: 4B2D). As a result, celastrol formed a covalent bond with the residue Cys^31^ in the crystal structure of PKM2 (Fig. 2B). To confirm Cys^31^ as the attachment site for celastrol, wild type PKM2 and mutant PKM2 (C31S) were parallelly incubated with celastrol-PEG3-biotin and detected by WB analysis. As a result, the mutant PKM2 (C31S) showed markedly less celastrol binding (Fig. S3). To examine the cellular celastrol-PKM2 conjugate, celastrol-PEG4-alkyne was synthesized as a small molecule probe. After the treatment with the probe or free celastrol, RAW264.7 cells were incubated with AFDye555-picolyl azide for the detection of the conjugate celastrol. In parallel, the cellular PKM2 was detected by immunostaining with primary anti-PKM2 antibody and fluorescent secondary antibody. As shown in Fig. 2C, the labeling of the conjugate celastrol was well co-localized with the fluorescence signal of PKM2 with the Pearson correlation coefficient of 0.85. These results indicated that celastrol could bind to the cellular PKM2. To examine the effect of celastrol on the enzyme activities of PKM1 and PKM2, recombinant PKM1 and PKM2 were respectively incubated with celastrol at several different concentrations. The enzyme activity was subsequently assayed by commercial kit. As shown in Fig. 2D, celastrol dose-dependently diminished the enzymatic activity of PKM2. Specifically, the covalent binding of celastrol caused the decline of PKM2 activity with the IC_50_ value of 0.305 mM. Interestingly, celastrol did not inhibit the activity of PKM1 even at the concentration of 0.5 mM (Fig. S2D). These results suggested that celastrol might selectively target PKM2.

### Celastrol induced metabolic reprogramming from glycolysis to oxidative phosphorylation in LPS-activated macrophages

**Figure 3:**
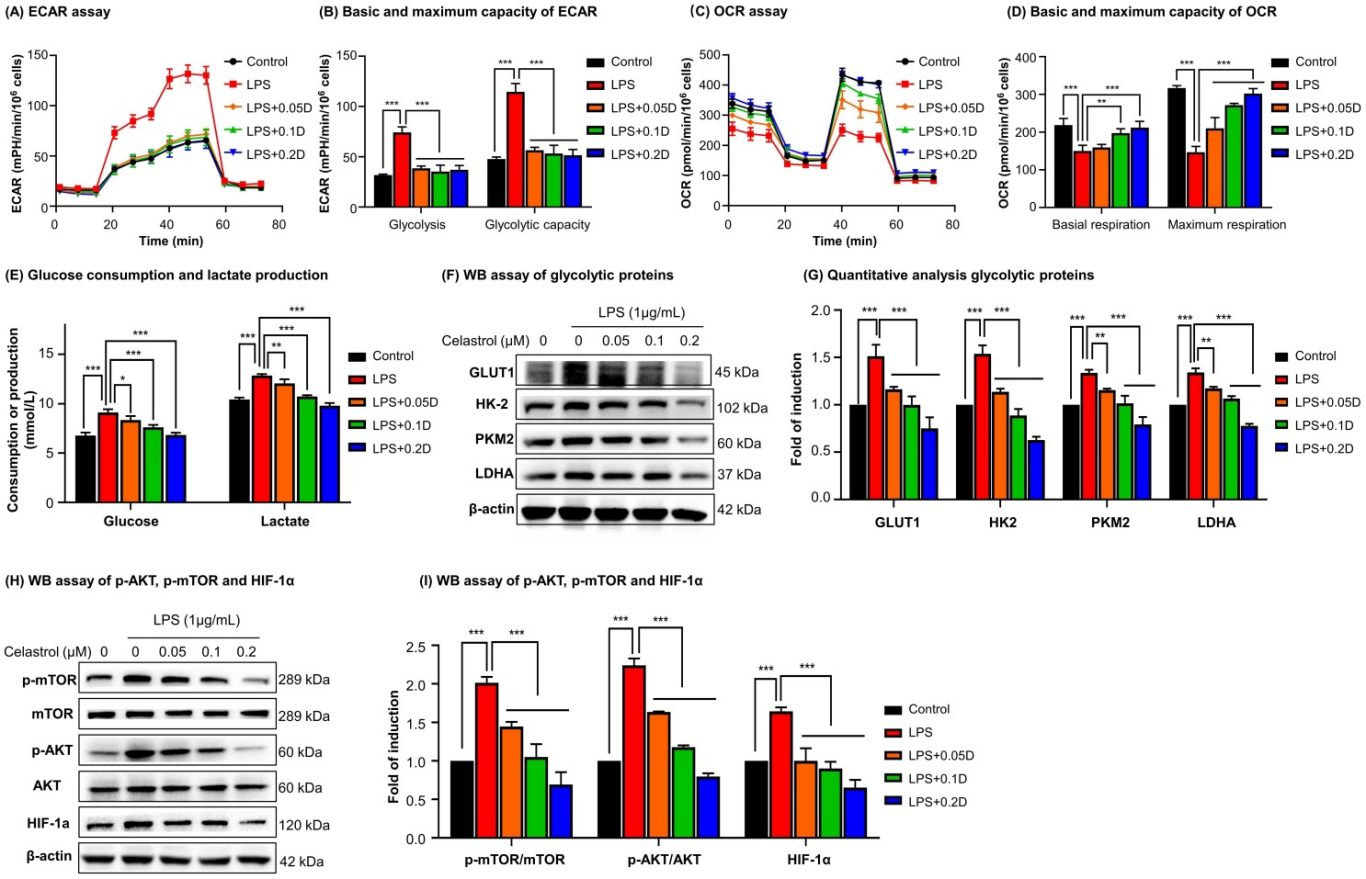
** Celastrol shifted macrophage metabolism from glycolysis to mitochondrial respiration against LPS stimulation. (A)** Assay for extracellular acidification rate (ECAR). **(B)** Quantification for the basic and maximum capacity of ECAR. **(C)** Assay for oxygen consumption rate (OCR). **(D)** Quantification for the basic and maximum capacity of OCR. Briefly, the cells were incubated with celastrol in the presence of LPS for 12 h. Assays for ECAR and OCR were performed with RAW264.7 cells according to Agilent Seahorse analysis guidelines. **(E)** Assays for glucose consumption and lactate production. The contents of glucose and lactate were measured using the commercial kits. **(F)** Inhibition of glycolytic proteins by celastrol. After treated celastrol and LPS as stated in “Methods”, RAW264.7 cells were lysed and examined by WB analysis. **(G)** Quantitative analysis results of glycolytic proteins (n=3). **(H)** Inhibition of glycolysis-regulating proteins (e.g., *p*-AKT, *p*-mTOR and HIF-1α) by celastrol. **(I)** Quantitative analysis results of glycolysis-regulating proteins (n=3). * *p* < 0.05, ** *p* < 0.01, ***p < 0.001 (LPS vs others).

To determine the effects of celastrol on metabolic status in macrophages, RAW264.7 cells were stimulated by LPS to undergo M1 polarization and co-treated with celastrol at indicated concentrations. As shown in Figs. 3A-3B, LPS stimulated aerobic glycolysis and glycolytic capacity, whereas celastrol significantly decreased the stimulatory effects of LPS even at the concentration of 0.05 μM. On the other hand, as shown in Figs. 3C-3D, LPS challenge reduced the basal and maximal respiration of RAW264.7 cells whereas celastrol dose-dependently antagonized the effects of LPS on OCR. These results showed that celastrol suppressed aerobic glycolysis while enhanced oxidative phosphorylation in LPS-induced macrophages. Secondly, glucose consumption and lactate level were determined by commercial kits. As shown in Fig. 3E, celastrol dose-dependently reduced glucose consumption and lactate production in LPS-stimulated macrophages. Thirdly, WB analysis confirmed that LPS increased the expression of several glycolytic enzymes (i.e., GLUT1, HK-2, PKM2, LDHA), whereas celastrol dose-dependently suppressed the stimulatory effects of LPS on glycolytic biomarkers (Figs. 3F-3G). Interestingly, neither LPS nor celastrol did affect the expression of PKM1 in RAW264.7 cells (Figs. S2B-C). Fourthly, we investigated the effects of celastrol on potential signaling pathways. As shown in Figs. 3H-3I, LPS increased the levels of p-AKT, p-mTOR and HIF-1α, whereas celastrol dose-dependently reduced the HIF-1α expression and the phosphorylation of AKT and mTOR. These results indicated that celastrol might inhibit Warburg effects through suppressing AKT-, mTOR- and HIF-1α-mediated signaling pathways.

### Celastrol inhibited the nuclear translocation of PKM2 and HIF-1α and skewed macrophage polarization from M1 to M2

**Figure 4:**
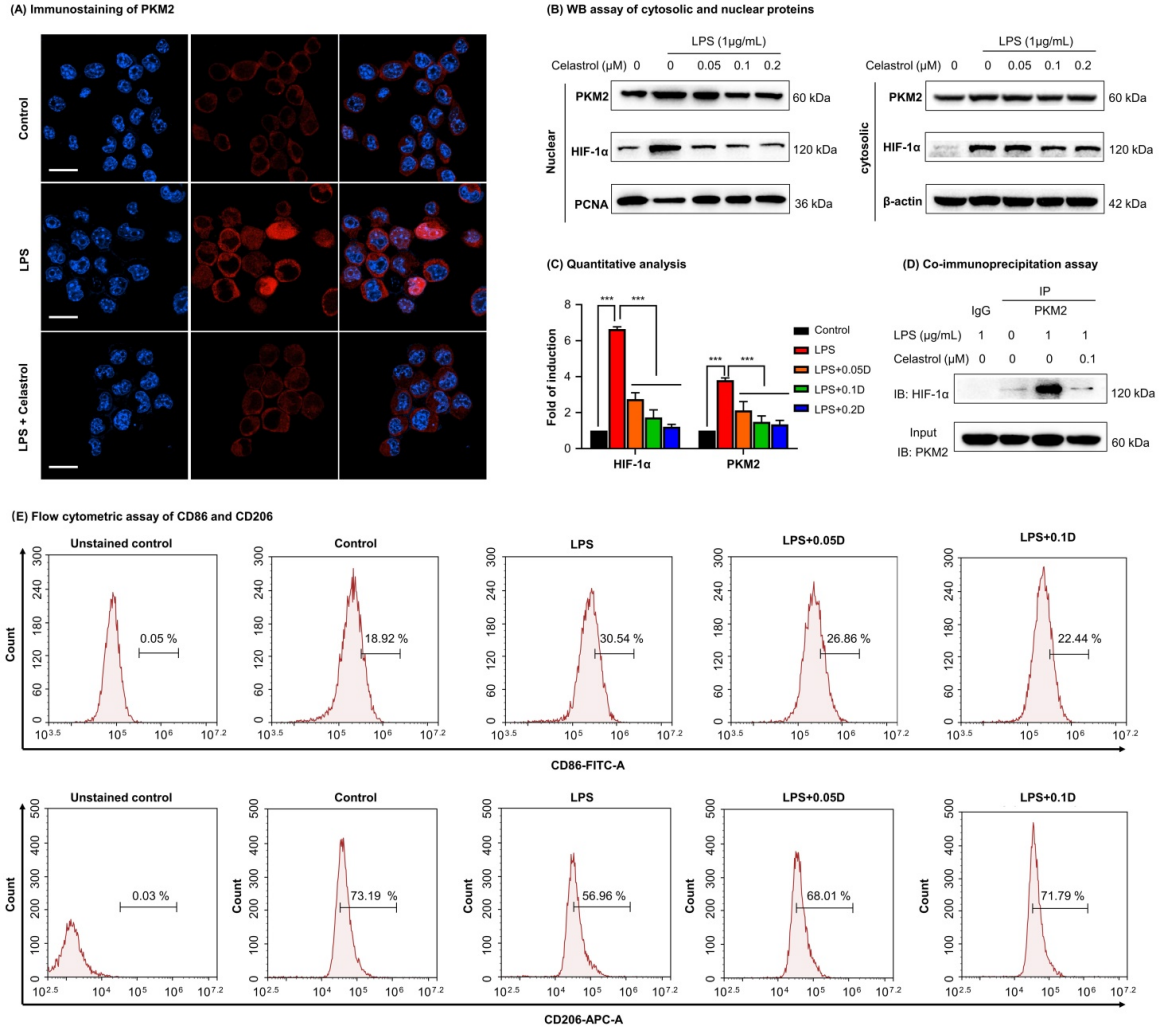
** Celastrol inhibited the nuclear translocation and interaction of PKM2 and HIF-1α while promoted phenotypic switch of macrophage polarization. (A)** Immunofluorescence imaging of cellular PKM2. After treated celastrol and LPS as stated in “Methods”, RAW264.7 cells were analyzed by immunofluorescence staining. Scale bar: 20 µm. **(B)** WB analysis of cytosolic and nuclear proteins (PKM2 and HIF-1α). **(C)** Quantitative analysis for the cytosolic and nuclear proteins. **(D)** Immunoprecipitation of PKM2-HIF-1α complex. Following drug treatment, RAW264.7 cells were lysed and subjected to immunoprecipitation with anti-PKM2 antibody, the bound proteins were analyzed by immunoblotting with anti-HIF-1α antibody. Input PKM2 in each group was immunoblotted as control. **(E)** Flow cytometric analysis of M1 macrophage biomarker CD86 and M2 macrophage biomarker CD206. ***p < 0.001 (LPS vs others).

To determine the effects of celastrol on PKM2 function, firstly, we examined the nuclear translocation of PKM2 by immunofluorescence staining. As a result, LPS upregulated PKM2 expression level and increased the nuclear translocation of PKM2 (Fig. 4A). Interestingly, celastrol reduced both the overall and the nuclear level of PKM2 expression. Secondly, we prepared and analyzed the expression levels of cytosolic and nuclear proteins. As results, LPS significantly promoted the nuclear levels of PKM2 and HIF-1α, whereas celastrol dose-dependently decreased the LPS-induced nuclear translocation of PKM2 and HIF-1α (Figs. 4B-4C). Thirdly, we performed immunoprecipitation assay to examine the effect of celastrol on the binding of PKM2 to HIF-1α. As a result, LPS promoted the binding of PKM2 with HIF-1α, whereas celastrol at the concentration of 0.1 μM abolished the formation of PKM2 with HIF-1α (Fig. 4D). Furthermore, to examine the potential relevance to macrophage polarization, we treated RAW264.7 cells with LPS and with or without celastrol, stained with M1 biomarker CD86 or M2 biomarker CD206 and analyzed the cells by flow cytometry. As shown in Fig. 4E, LPS increased CD86 expression in macrophages from 18.92% (untreated control) to 30.54%, whereas celastrol dose-dependently decreased the population of CD86^+^ cells. On the other hand, LPS reduced the population of CD206^+^ macrophages from 73.19% (untreated control) to 56.96%, whereas celastrol dose-dependently increased the population of CD86^+^ cells. These results indicated that celastrol suppressed the nuclear translocation of PKM2 and HIF-1α and skewed the phenotypic switch of macrophages from M1- to M2-type.

### Celastrol ameliorated HFD-induced NAFLD in C57BL/6 mice

**Figure 5:**
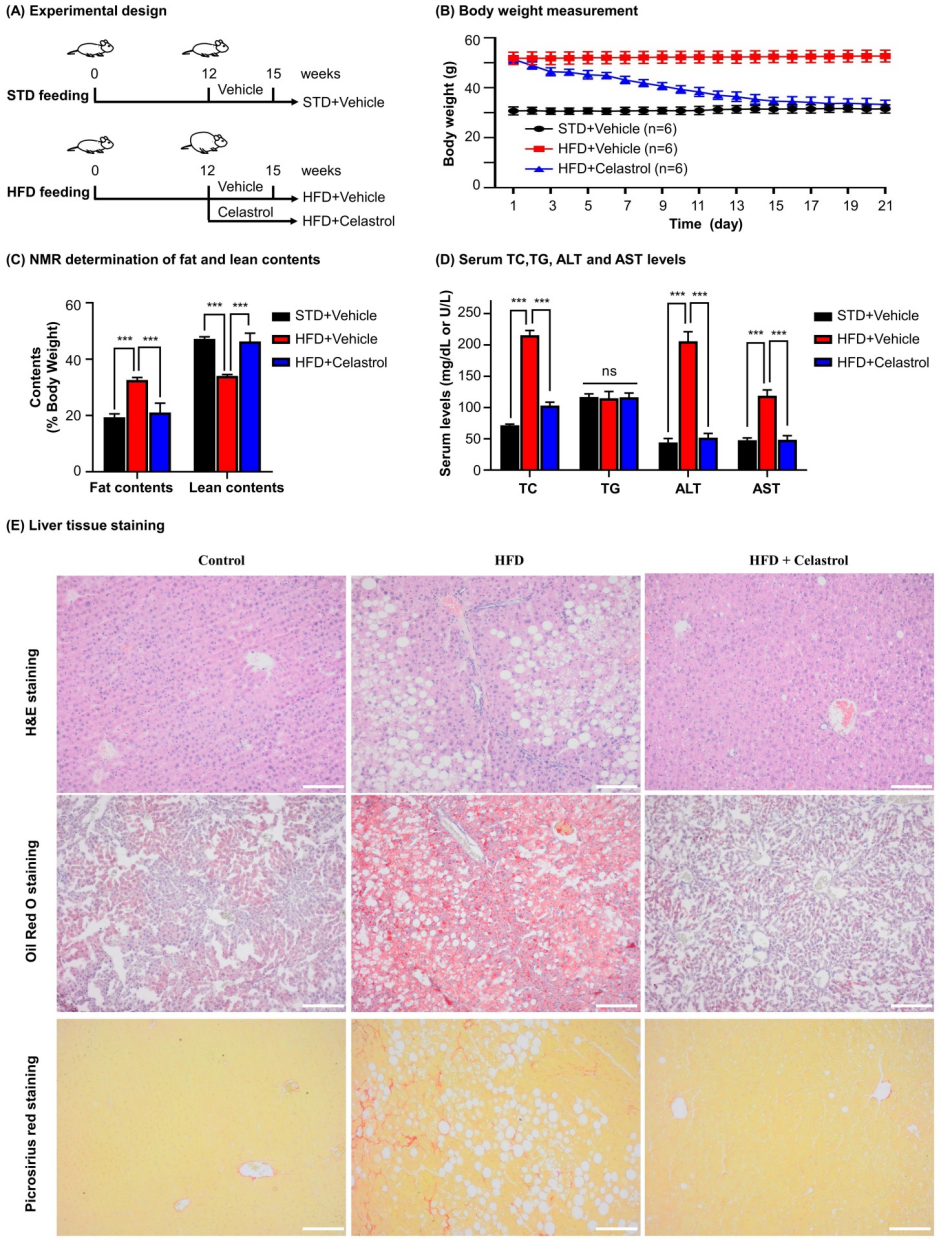
***In vivo* evaluation of celastrol against HFD-induced NAFLD in C57BL/6 mice. (A)** Experimental design. **(B)** Monitoring of body weight. The mice were weighed daily after receiving drug (n=6). **(C)** NMR determination of fat contents and lean contents. On the final day of drug administration, mice were analyzed for the fat contents and lean contents as stated in “Methods”. **(D)** Assay for the serum levels of TC (mg/dL), TG (mg/dL), ALT (U/L) and AST (U/L). The results were expressed as Mean ± SD. **(E)** Staining of liver tissues. After drug treatment, mouse liver tissues were collected and examined by H&E staining (Upper Panel), Oil Red O staining (Middle Panel) and Picrosirius Red staining (Bottom Panel). Scale bar of Upper Panel and Bottom Panel: 100 µm, Scale bar of Middle Panel: 200 µm. * *p* < 0.05, ** *p* < 0.01, ***p < 0.001 (HFD vs others).

To investigate the efficacy of celastrol on NAFLD mice, we fed C57BL/6 mice with HFD to induce NAFLD and subsequently administrated with celastrol (Fig. 5A). Firstly, as shown in Fig. 5B, celastrol markedly reduced the body weight of NAFLD mice. Secondly, as shown in Fig. 5C, celastrol profoundly decreased the fat contents of NAFLD mice while recovered the lean contents. Thirdly, as shown in Fig. 5D, celastrol reduced serum contents of TC, AST and ALT in NAFLD mice except for the serum TG levels. Fourthly, the effects of celastrol on hepatic lipid deposition and inflammatory cell infiltration were evaluated by staining with H&E stain, Oil Red O dye and Picrosirius Red stain. As shown in Fig. 5E, H&E staining showed that HFD greatly caused adipocyte hypertrophy and inflammatory cells infiltration in the liver, whereas celastrol effectively attenuated hepatic adipocyte hypertrophy and inflammatory cell infiltration. In addition, Oil Red O staining confirmed that celastrol ameliorated lipid deposition in liver. Picrosirius Red staining indicated that HFD promoted the synthesis of deposition of collagen in liver, whereas celastrol eliminated the deposition of collagen. These results showed that celastrol reduced adipose hypertrophy, inflammatory cells infiltration and hepatic fibrosis in the NAFLD mice.

### Celastrol skewed hepatic macrophage M1/M2 polarization in NAFLD mice

**Figure 6:**
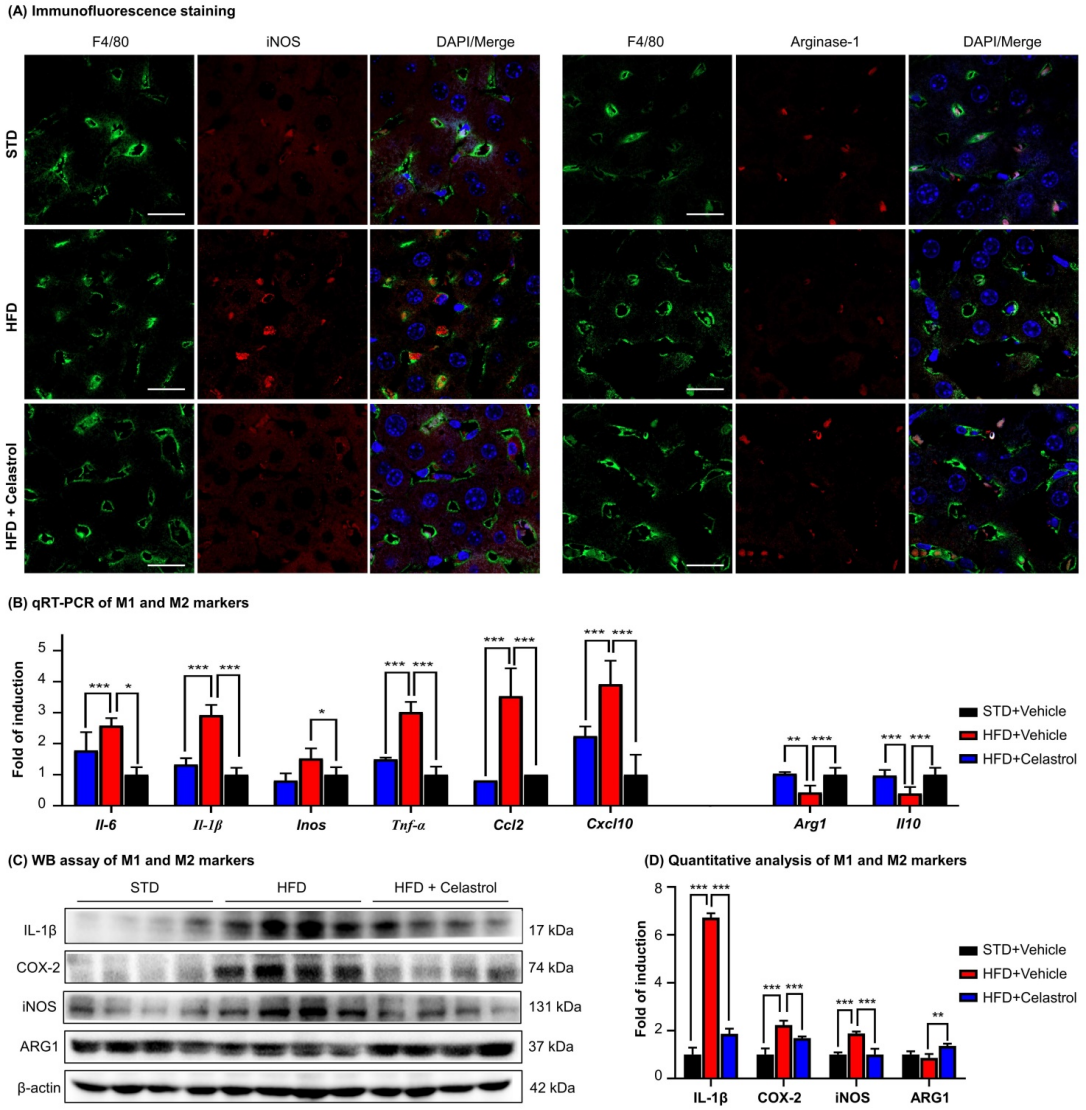
** Celastrol skewed hepatic macrophage M1/M2 polarization in NAFLD mice. (A)** Detection of iNOS and arginase-1 in livers. F4/80 was stained as macrophage biomarker. The cell nuclei were detected with DAPI. Scale bar: 20 µm. **(B)** qRT‐PCR quantification of M1 macrophage biomarkers and M2 macrophage biomarkers. **(C)** WB analysis of macrophage polarization biomarkers (n=4). **(D)** Quantitative analysis of macrophage polarization biomarkers. * *p* < 0.05, ** *p* < 0.01, ***p < 0.001 (HFD vs others).

To examine the effects of celastrol on macrophages polarization in NAFLD mice, we detected a panel of macrophage polarization biomarkers and inflammatory biomarkers. Firstly, HFD increased the number of iNOS^+^ M1 macrophages, while decreased the number of ARG1^+^ M2 macrophages. Interestingly, celastrol reduced the number of iNOS^+^ M1 macrophages, while increased the number of ARG1^+^ M2 macrophages (Fig. 6A). Secondly, based on qRT-PCR analysis for pro-inflammatory macrophage M1 biomarkers (i.e., iNOS, IL-6, IL-1β, TNF-α, CCL-2, CXCL-10) and anti-inflammatory macrophage M2 biomarkers (i.e., ARG1, IL-10) in Fig. 6B, celastrol markedly reduced the HFD-induced the upregulation of macrophage M1 biomarkers, while elevated the levels of macrophage M2 biomarkers. Thirdly, according to WB analysis for several representative macrophage M1/M2 biomarkers in Figs. 6C-6D, celastrol remarkably ameliorated HFD-disrupted expression of macrophage M1/M2 biomarkers at the protein level. These results suggested that celastrol skewed hepatic macrophage M1/M2 polarization in NAFLD mice.

### Celastrol inhibited Warburg effect via targeting PKM2 in NAFLD mice

**Figure 7:**
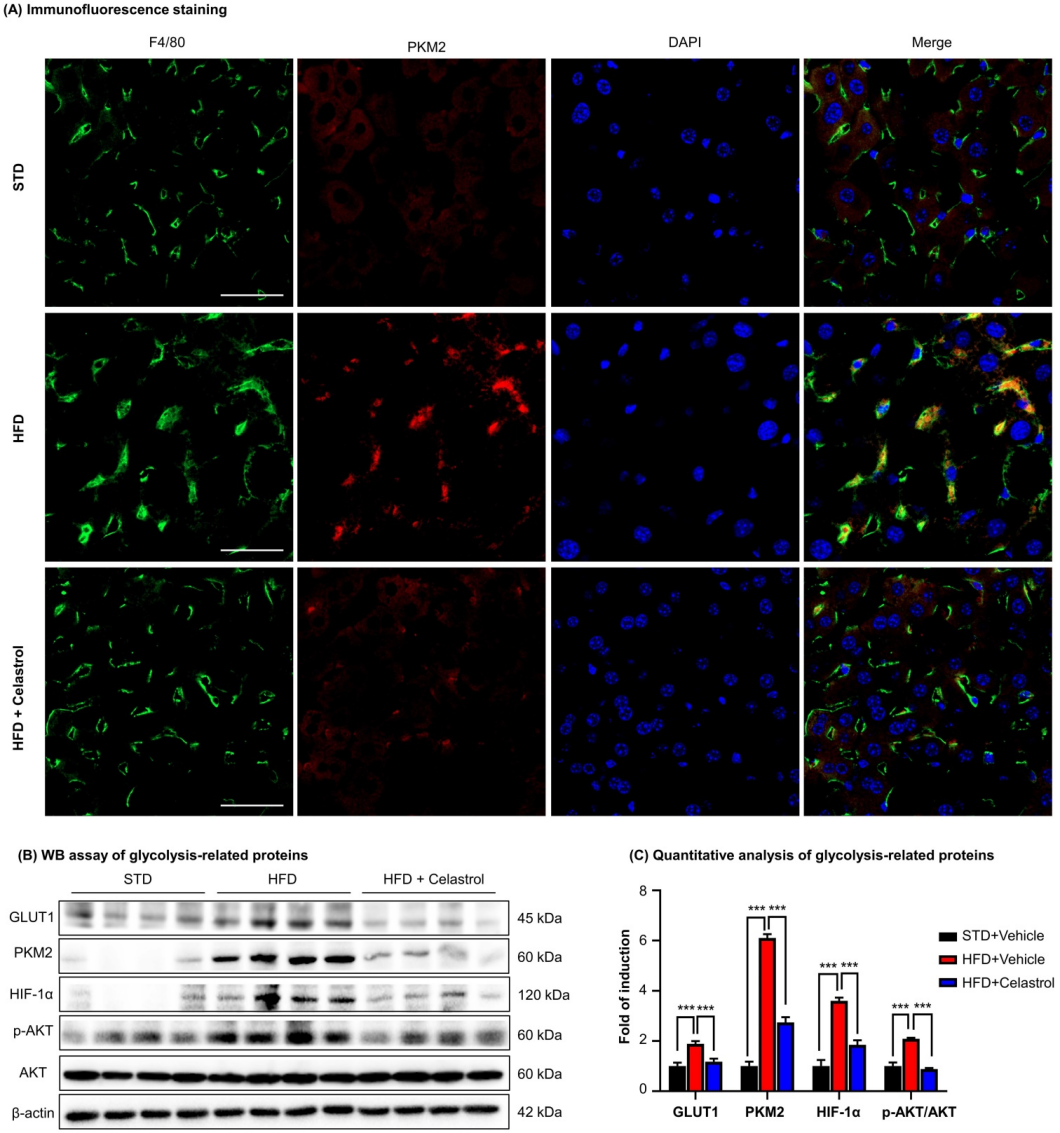
** Celastrol inhibited Warburg effect via targeting PKM2 in NAFLD mice. (A)** Detection of PKM2 in livers. Scale bar: 40 µm. **(B)** WB analysis of glycolysis regulators. **(C)** Quantitative analysis of glycolysis regulators. * *p* < 0.05, ** *p* < 0.01, ***p < 0.001 (HFD vs others).

To further investigate the effects of celastrol on the metabolism and glycolysis-related signaling pathway in NAFLD mice, firstly, we employed immunofluorescence staining to examine the expression of PKM2 in hepatic macrophages in NAFLD mice. As shown in Fig. 7A, PKM2 was greatly expressed in the livers, especially in hepatic macrophages of NAFLD mice, whereas celastrol treatment effectively decreased PKM2 expression in hepatic macrophages. Secondly, the *in vivo* effects of celastrol on the expression of glycolysis-related proteins (i.e., PKM2, p-AKT, GLUT1 and HIF-1α) were examined by WB analysis. As shown in Figs. 7B-7C, celastrol remarkably suppressed the HFD-induced increase of expression of glycolysis-related enzymes and signaling proteins. These results consistently supported that celastrol might ameliorate the pathological alterations in NAFLD mice through inhibiting Warburg effect and skewing macrophage M1/M2 polarization through targeting PKM2.

## Discussion

Macrophage polarization has recently emerged as an important therapeutic target against various metabolic diseases including insulin resistance, NAFLD and atherosclerosis 35. Our previous studies demonstrated that natural product celastrol effectively skewed macrophage M1/M2 polarization in experimental obesity 28. Here, we identified PKM2 as a molecular target for celastrol through an affinity pulldown procedure as outlined in Fig. 1A. PKM2 is one of the regulatory enzymes in glycolysis and appears to be highly associated with Warburg effect in the proliferating cancer cells and inflammatory immune cells 14. These results stimulated us to hypothesize that celastrol might induce the reprogramming of metabolism and inflammatory response via covalent modification of PKM2. Thus, celastrol may be a potential drug candidate for the resolution of inflammation and the amelioration of adipocyte hypertrophy in the mouse model of NAFLD.

PKM2 is well-known to form dimer or tetramer in response to endogenous and exogenous stimuli 36. PKM2 tetramer at the compact state (R-state) exhibits high PK activity for transferring of phosphate from phosphoenolpyruvate (PEP) to ADP, whereas PKM2 tetramer at the loose state (T-state) is less stable and likely collapses into PKM2 dimer/monomer. PKM2 dimer/monomer exhibits low PK activity and tends to translocate to the nucleus and regulate gene transcription. Importantly, several allosteric regulators bind to PKM2, induce the changes in the spatial conformation and the electrostatic force of the protein, and further affect the transition state of PKM2 36. As an example, phenylalanine could stabilize the inactive T-state tetrameric conformer and inhibit PKM2 (M2PYK) activity, whereas thyroid hormone (triiodo-L-thyronine, T3) stabilized the inactive PKM2 monomer 37. Such unique adaptive capacity of PKM2 is also tightly regulated by three cysteine residues (i.e., Cys^31^, Cys^424^ and Cys^358^) to show different spatial conformations and enzymatic activity 38. Under oxidative stress, intracellular reactive oxygen species (ROS) induces the oxidation of Cys^358^ and diminishes PKM2 activity, triggering pro-survival signals in cancer cells 38. Several synthetic and natural compounds products (e.g., gliotoxin, resveratrol, shikonin, vitamin K, naphthoquinone C3k and C3f) exhibit anti-cancer activity via inhibiting PKM2 and modulating glycolysis 39-42. These small molecules may inhibit PKM2 activity via non-covalent or covalent binding to the active site of PKM2. Small molecules including shikonin and synthetic benzoxepane derivatives could suppress the proliferation of cancer cells and regulate macrophage polarization via covalent binding to PKM2 16, 43. Plant-derived micheliolide covalently bound to Cys^424^, promoted the formation of PKM2 tetramer and suppressed the cancer cells growth and tumorigenesis 44. On the other hand, celastrol is well-documented for suppressing inflammation and lipid accumulation via regulating macrophage polarization 21. Celastrol bears quinone methide moiety and may readily form a covalent bond with the cysteine residues in various proteins including Cdc37, IKKβ, Yap1 and HSP90 co-chaperone p23 29. In this study, firstly, PKM2 was determined to be a predominant celastrol-bound protein from RAW264.7 cells through affinity pulldown with celastrol-coated agarose resin (Fig. 1B), while celastrol could not bind to the cellular PKM1 protein (Fig. S2A). Secondly, we prepared recombinant mouse PKM2 protein and verified the covalent binding of celastrol to PKM2. In practice, a biotin tag was incorporated to celastrol to yield celastrol-PEG3-biotin as small molecule probe. In the presence of excessive un-tagged celastrol, the detection of celastrol-PEG3-biotin was fully prevented, proving the formation of covalent conjugate (Fig. 1C). Thirdly, peptide mapping revealed that celastrol formed covalent conjugate with the residue Cys^31^ (Figs. 2A-2B), whereas mutated PKM2 (C31S) showed markedly less celastrol binding (Fig. S3). Fourthly, small molecule probe celastrol-PEG3-alkyne was consistently colocalized with PKM2 in RAW264.7 cells (Fig. 2C). Fifthly, celastrol concentration-dependently inhibited PKM2 activity (Fig. 2D) while did not affect PKM1 activity (Fig. S2D). Celastrol appeared to support the formation of PKM2 tetramer in LPS-treated RAW264.7 cells (Fig. S4). Covalent modification of Cys^31^ by celastrol may alter the spatial conformation of PKM2, trap PKM2 tetramer in the inactive T-state and possibly affect the binding of PEP to PKM2. Collectively, these results confirmed that celastrol could be a covalent inhibitor for PKM2.

PKM2 is the key target to regulate the cellular metabolism and inflammatory response 15. The PKM2 activity is an index of Warburg effect and determines the phenotypes and functions of macrophages within the specific pathological context. PKM2 is activated to increase glycolysis in tumors and LPS-stimulated macrophages whereas inhibition of PKM2 might reduce lactate production and alleviate inflammation 14. Hyperactive PKM2 facilitates aerobic glycolysis to generate energy and spare lipids in the storage in M1 macrophages. In contrast, inhibition of PKM2 forces macrophages to switch aerobic glycolysis to mitochondrial oxidative phosphorylation to produce ATP and undergo M2 polarization 9, 45. In the present study, we employed LPS to stimulate macrophage M1 polarization and induce inflammatory response. Subsequently, celastrol was evaluated for the effects on glycolysis, mitochondrial respiration, inflammatory biomarkers and macrophage polarization biomarkers. Indeed, celastrol effectively reduced glucose uptake, lactate production and the expression of pivotal glycolytic proteins (i.e., GLUT1, HK-2, PKM2, LDHA) while promoted the mitochondrial respiration in LPS-challenged RAW264.7 cells (Fig. 3). Accordingly, flow cytometric analysis confirmed that celastrol skewed macrophage polarization from inflammatory M1 phenotype to anti-inflammatory M2 phenotype in LPS-challenged RAW264.7 cells (Fig. 4E). As for molecular mechanisms, the Akt and mTOR signaling pathways are implicated in regulating macrophage metabolism and activation 46. Akt activation upregulates several glycolytic enzymes (e.g., GLUT1, PFKFB3 and LDHA) and thereby enhances glucose utilization 10. The activation of the Akt-mTOR axis modulates multiple downstream effectors to govern macrophage polarization 46-49. Moreover, transcription factor HIF1α also has significant role in regulating glycolysis through the transcriptional induction of several glycolytic enzymes 50. Interestingly, PKM2 could translocate to the nucleus, form complex with HIF1α and facilitate HIF1α to regulate the expression of inflammatory factors and glycolytic enzymes 15. Several previous studies demonstrated that celastrol could regulated macrophage functions by suppressing the activation of NF-κB and MAPKs and/or promoting macrophage M2 polarization 28, 51, 52. The present study showed that celastrol reduced LPS-induced activation of ATK, mTOR and HIF1α (Figs. 3H -3I), suppressed the LPS-induced nuclear translocation of PKM2 and HIF1α (Figs. 4A, 4B-4C) and prevented LPS-induced formation of PKM2-HIF1α complex (Fig. 4D). These results indicated that celastrol might induce metabolic reprogramming and skew macrophage M1/M2 polarization via targeting the cross-talks among the Akt-mTOR-HIF1α-PKM2 pathways.

Macrophages play significant roles in the onset and progression of NAFLD 5. Our *in vitro* studies indicated that covalent inhibition of PKM2 might be a key mechanism underlying the role of celastrol on the reprogramming of metabolic networks and inflammatory response in macrophages. It is important to evaluate the pharmacological potential of celastrol for treating NAFLD. Indeed, NAFLD mouse model was induced by feeding mice with HFD for three months and subsequently received celastrol for consecutive 21 days as outlined in Fig. 5A. We found that celastrol reduced body weight in a time-dependent manner (Fig. 5B). NMR determination showed that celastrol reduced body fat while increased lean mass (Fig. 5C). Moreover, celastrol effectively ameliorated the histopathological alterations of NAFLD. Specifically, celastrol decreased the serum levels of cholesterol, ALT and AST, hepatic lipid accumulation, inflammatory cells infiltration and hepatic fibrosis (Figs. 5D-5E). Furthermore, the effects of celastrol on macrophage polarization were examined in NAFLD mice in a great detail. Celastrol effectively reduced the expression of inflammatory M1 macrophage biomarkers while enhanced the expression of anti-inflammatory M2 macrophage biomarkers (Figs. 6A-C). The immunofluorescence staining results further indicated that celastrol effectively reduced the protein level of PKM2 in hepatic macrophages (Fig. 7A). WB analysis confirmed that celastrol decreased the expression of glycolytic enzymes (i.e., GLUT1, PKM2) and the key signaling proteins (i.e., p-Akt, HIF1α) in liver of NAFLD mice (Figs. 7B-7C). These results consistently supported that celastrol might ameliorate the pathological alterations in NAFLD mice through inhibiting Warburg effect and skewing macrophage M1/M2 polarization in Akt- and HIF1α-dependent manner.

## Conclusion

Covalent inhibition of PKM2 effectively suppressed Warburg effect in inflammatory macrophages and skewed macrophages M1/M2 polarization. The key finding was that celastrol induces metabolic and inflammatory reprogramming via covalently binding to PKM2, thereby parallelly suppressing glycolysis and inhibiting the activities of the PKM2-HIF-1α signaling pathway in the induction of glycolytic enzymes and inflammatory mediators. Therefore, the covalent inhibition of PKM2 is a new mechanism of celastrol to reprogram metabolic and inflammatory pathways in hepatic macrophages against NAFLD/NASH.

## Supplementary Material

Supplementary figures and table.

## Abbreviations

ALT: Alanine transaminase
AST: Aspartate transaminase
BOP: (benzotriazol-1-yloxy) tris (dimethylamino) phosphonium hexafluorophosphate
CCMR: Centre for Comparative Medicine Research
DMEM: Dulbecco's Modified Eagle Medium
DMF: Dimethylformamide
DCM: dichloromethane
2-DG: 2-deoxy-glucose
ER: endoplasmic reticulum
ECL: enhanced chemiluminescence
ECAR: extracellular acidification rate
FBS: fetal bovine serum
HIF1α: hypoxia-inducible factor 1α
HFD: high fat diet
H&E: Hematoxylin and eosin
IPTG: isopropyl β-D-1-thiogalactopyranoside
LPS: Lipopolysaccharides
NAFLD: non-alcoholic fatty liver disease
NASH: non-alcoholic steatohepatitis
OCR: oxygen consumption rate
PKM2: pyruvate kinase M2
PKM1: pyruvate kinase M1
PVDF: polyvinylidene difluoride
PFA: paraformaldehyde
PEP: phosphoenolpyruvate
qRT-PCR: quantitative real-time polymerase chain reaction
ROS: reactive oxygen species
TC: cholesterol
TG: triglyceride
WB: western blot
