# Supplementary material for: Covalent Inhibition of Pyruvate Kinase M2 Reprograms Metabolic and Inflammatory Pathways in Hepatic Macrophages against Non-alcoholic Fatty Liver Disease
Source: Int J Biol Sci. 2022 Aug 15;18(14):5260–75. doi: 10.7150/ijbs.73890 (PMC9461663; doi:10.7150/ijbs.73890)
Supplement: Supplementary file 1 — Supplementary figures and table. [file ijbsv18p5260s1.pdf]

Table S1. Key Resources Table

Reagent type (species) or resource	Designation	Source or reference	Identifiers	Additional information
Antibody	Anti-PKM2 (rabbit monoclonal)	Cell Signaling Technology (Danvers, MA, USA)	Cat. #: 4053s	WB (1:1000) IF (1:500)
Antibody	Anti-PKM1 (rabbit polyclonal)	Thermo Fisher Scientific (Waltham, MA, USA)	Cat. #: PA5-120660	WB (1:1000)
Antibody	Anti-GLUT1 (rabbit monoclonal)	Cell Signaling Technology (Danvers, MA, USA)	Cat. #: 12939S	WB (1:1000)
Antibody	Anti-HK2 (rabbit polyclonal)	Abclonal (Cambridge, MA, USA)	Cat. #: A0994	WB (1:1000)
Antibody	Anti-LDHA (rabbit polyclonal)	Cell Signaling Technology (Danvers, MA, USA)	Cat. #: 2012S	WB (1:1000)
Antibody	Anti-β-actin (rabbit polyclonal)	Thermo Fisher Scientific (Waltham, MA, USA)	Cat. #: PA1-183	WB (1:1000)
Antibody	Anti-Phospho-mTOR (rabbit monoclonal)	Cell Signaling Technology (Danvers, MA, USA)	Cat. #: 5536S	WB (1:1000)
Antibody	Anti-mTOR (rabbit monoclonal)	Cell Signaling Technology (Danvers, MA, USA)	Cat. #: 2983S	WB (1:1000)
Antibody	Anti-Phospho-Akt (rabbit monoclonal)	Cell Signaling Technology (Danvers, MA, USA)	Cat. #: 4058S	WB (1:1000)
Antibody	Anti-Akt (rabbit monoclonal)	Cell Signaling Technology (Danvers, MA, USA)	Cat. #: 4685S	WB (1:1000)

Antibody	Anti-HIF-1α (mouse monoclonal)	Thermo Fisher Scientific (Waltham, MA, USA)	Cat. #: MA116504	WB (1:1000)
Antibody	Anti-PCNA (mouse monoclonal)	Thermo Fisher Scientific (Waltham, MA, USA)	Cat. #: 14-9910-82	WB (1:1000)
Antibody	FITC-conjugated anti-CD86 (mouse monoclonal)	eBioscience (San Diego, CA, USA)	Cat. #: 14-9910-82	Flow (1:200)
Antibody	APC-conjugated anti-CD206 (mouse monoclonal)	eBioscience (San Diego, CA, USA)	Cat. #: 17-2061-82	Flow (1:200)
Antibody	Anti-F4/80 (rat monoclonal)	Abcam (Cambridge, United Kingdom)	Cat. #: ab6640	IF (1:200)
Antibody	Anti-iNOS (rabbit polyclonal)	Thermo Fisher Scientific (Waltham, MA, USA)	Cat. #: PA1-036	IF (1:200)<br>WB (1:1000)
Antibody	Anti-Arginase-1 (rabbit polyclonal)	Santa Cruz Biotechnology (Dallas, TX, USA)	Cat. #: sc20150	IF (1:100)<br>WB (1:500)
Antibody	Anti-IL-1β (mouse monoclonal)	Thermo Fisher Scientific (Waltham, MA, USA)	Cat. #: 14-7012-81	WB (1:1000)
Antibody	Anti-COX-2 (rabbit monoclonal)	Cell Signaling Technology (Danvers, MA, USA)	Cat. #: 12282S	WB (1:1000)
Antibody	Anti-rabbit IgG (H+L) Alexa Fluor 594 (goat polyclonal)	Thermo Fisher Scientific (Waltham, MA, USA)	Cat. #: A-11012	IF (1:500)
Antibody	Anti-rat IgG (H+L) Alexa Fluor 488 (goat polyclonal)	Thermo Fisher Scientific (Waltham, MA, USA)	Cat. #: A-11006	IF (1:500)

Antibody	Anti-rabbit IgG (H+L) Alexa Fluor 488 (goat polyclonal)	Thermo Fisher Scientific (Waltham, MA, USA)	Cat. #: A-11008	IF (1:500)
Antibody	Anti-rabbit HRP-conjugated IgG secondary antibody (goat polyclonal)	Sigma-Aldrich (St. Louis, MO, USA)	Cat. #: A0545	WB (1:10000)
Antibody	Anti-mouse HRP-conjugated IgG secondary antibody (Horse)	Cell Signaling Technology (Danvers, MA, USA)	Cat. #: 7076S	WB (1:10000)
Antibody	HRP-Conjugated Streptavidin	Thermo Fisher Scientific (Waltham, MA, USA)	Cat. #: N100	WB (1:10000)
Compound，biological reagent	Celastrol	Nanjing Spring and Autumn Biological Engineering Co., Ltd. (Nanjing, Jiangsu, China).		Purity ＞98% (HPLC)
Compound，biological reagent	Biotin-PEG3-amine	Hunan Huateng Pharmaceutical Company (Changsha, Hunan, China)	Cat. #: 11025	
Compound，biological reagent	Alkyne-PEG4-amine	Hunan Huateng Pharmaceutical Company (Changsha, Hunan, China)	Cat. #: 10749	
Compound，biological reagent	Azide-PEG3-amine	Hunan Huateng Pharmaceutical Company (Changsha, Hunan, China)	Cat. #: 10610	
Compound，biological reagent	AFDye555-picolyl azide	Click Chemistry Tools (Scottsdale, AZ, USA)	Cat. #: 1288-5	
Compound，biological reagent	Dulbecco's Modified Eagle Medium (DMEM)	Thermo Fisher Scientific (Waltham, MA, USA)	Cat. #: 12800082	

Compound, biological reagent	Fetal bovine serum (FBS)	Thermo Fisher Scientific (Waltham, MA, USA)	Cat. #: 10270106	
Compound, biological reagent	Penicillin-Streptomycin	Thermo Fisher Scientific (Waltham, MA, USA)	Cat. #: 15140122	
Compound, biological reagent	Lipopolysaccharides from Escherichia coli O111:B4	Sigma-Aldrich (St. Louis, MO, USA)	Cat. #: L4391	LPS
Compound, biological reagent	DAPI	Thermo Fisher Scientific (Waltham, MA, USA)	Cat. #: D1306	
Compound, biological reagent	DSS (disuccinimidyl suberate)	Thermo Fisher Scientific (Waltham, MA, USA)	Cat. #: 21555	
Compound, biological reagent	RIPA buffer	Sigma-Aldrich (St. Louis, MO, USA)	Cat. #: R0278	
Compound, biological reagent	High fat diet (HFD)	Research Diets, Inc (New Brunswick, NJ, USA)	Cat. #: D12492	
Compound, biological reagent	Hematoxylin Solution	Sigma-Aldrich (St. Louis, MO, USA)	Cat. #: HHS32-1L	
Compound, biological reagent	Eosin Y-solution 0.5% alcoholic	Sigma-Aldrich (St. Louis, MO, USA)	Cat. #: 1.02439	
Compound, biological reagent	Oil Red O	Sigma-Aldrich (St. Louis, MO, USA)	Cat. #: O0625	
Compound, biological reagent	Sirius Red	Sigma-Aldrich (St. Louis, MO, USA)	Cat. #: 365548	
Commercial assay or kit	Click-&-Go Dde Protein Enrichment Kit	Click Chemistry Tools (Scottsdale, AZ, USA)	Cat. #: 1152	
Commercial assay or kit	Pyruvate Kinase Activity Assay Kit	BioVision (Milpitas, CA, USA)	Cat. #: K709	

Commercial assay or kit	Seahorse XF Glycolysis Stress Test Kit	Agilent Technologies (Santa Clara, CA, USA)	Cat. #: 103020-100
Commercial assay or kit	Seahorse XF Cell Mito Stress Test Kit	Agilent Technologies (Santa Clara, CA, USA)	Cat. #: 103015-100
Commercial assay or kit	Glucose assay kit	Nan Jing Jian Cheng Bioengineering Inc.(Nanjing, Jiangsu, China)	Cat. #: A154-1-1
Commercial assay or kit	Lactic Acid assay kit	Nan Jing Jian Cheng Bioengineering Inc.(Nanjing, Jiangsu, China)	Cat. #: A019-2-1
Commercial assay or kit	NE-PER Nuclear and Cytoplasmic Extraction Reagents	Thermo Fisher Scientific (Waltham, MA, USA)	Cat. #: 78835
Commercial assay or kit	Protein A/G PLUS-Agarose	Santa Cruz Biotechnology (Dallas, TX, USA)	Cat. #: sc-2003
Commercial assay or kit	Cytofix/Cytoperm ™ Fixation/Permeabilization Kit	BD Biosciences (San Diego, CA, USA)	Cat. #: 554714
Commercial assay or kit	Pierce™ BCA Protein Assay Kit	Thermo Fisher Scientific (Waltham, MA, USA)	Cat. #: 23227
Commercial assay or kit	Bio-Rad Protein Assay Dye Reagent Concentrate	Bio-Rad Laboratories (Hercules, CA, USA)	Cat. #: 5000006
Commercial assay or kit	Amersham™ ECL™ Select Western Blotting Detection Reagent	GE Healthcare (Chicago, IL, USA)	Cat. #: RPN2235
Commercial assay or kit	Total cholesterol (TC) assay kit	Stanbio Laboratory (Boerne, TX, USA)	Cat. #: 1010

Commercial assay or kit	Triglyceride (TG) assay kit	Stanbio Laboratory (Boerne, TX, USA)	Cat. #: 2100	
Commercial assay or kit	Aspartate transaminase (AST) assay kit	Stanbio Laboratory (Boerne, TX, USA)	Cat. #: 2920	
Commercial assay or kit	Alanine transaminase (ATL) assay kit	Stanbio Laboratory (Boerne, TX, USA)	Cat. #: 2930	
Commercial assay or kit	Mut Express II Fast Mutagenesis Kit V2	Nanjing Vazyme Biotech Co., Ltd (Nanjing, Jiangsu, China)	Cat. #: C214	
Commercial assay or kit	TRIzol™ Reagent	Thermo Fisher Scientific (Waltham, MA, USA)	Cat. #: 15596026	
Commercial assay or kit	RevertAid first-strand cDNA synthesis kit	Thermo Fisher Scientific (Waltham, MA, USA)	Cat. #: K1622	
Commercial assay or kit	SYBR Green mix	QIAGEN (Valencia, CA USA)	Cat. #: 204056	
Sequence-based reagent	Mm_Il6_1_SG	QIAGEN (Valencia, CA USA)	Cat. #: QT00098875	*Il-6*
Sequence-based reagent	Mm_Il1b_2_SG	QIAGEN (Valencia, CA USA)	Cat. #: QT01048355	*Il-1β*
Sequence-based reagent	Mm_Nos2_1_SG	QIAGEN (Valencia, CA USA)	Cat. #: QT00100275	*Inos*
Sequence-based reagent	Mm_Tnf_1_SG	QIAGEN (Valencia, CA USA)	Cat. #: QT00104006	*Tnf-α*
Sequence-based reagent	Mm_Ccl2_1_SG	QIAGEN (Valencia, CA USA)	Cat. #: QT00167832	*Ccl2*
Sequence-based reagent	Mm_Cxcl10_1_SG	QIAGEN (Valencia, CA USA)	Cat. #: QT00093436	*Cxcl10*

Sequence-base d reagent	Mm_Arg1_1_SG	QIAGEN (Valencia, CA USA)	Cat. #: QT00134288	*Arg1*
Sequence-base d reagent	Mm_Il10ra_1_SG	QIAGEN (Valencia, CA USA)	Cat. #: QT00112742	*Il10*
Recombinant DNA reagent	pET-28a	Merck Limited (Kenilworth, NJ, USA)	Cat. #: 69864	
Recombinant DNA reagent	pLHCX-Flag-mPK M2	Addgene (Watertown, MA, USA)	Cat. #: 42512	
Recombinant DNA reagent	pLHCX-Flag-mPK M1	Addgene (Watertown, MA, USA)	Cat. #: 42511	
Biochemical reagent	XhoI	New England Biolabs (Ipswich, MA, United States)	Cat. #: R0146S	
Biochemical reagent	EcoRI	New England Biolabs (Ipswich, MA, United States)	Cat. #: R0101S	
Biochemical reagent	T4 DNA ligase	New England Biolabs (Ipswich, MA, United States)	Cat. #: M0202S	

Figures

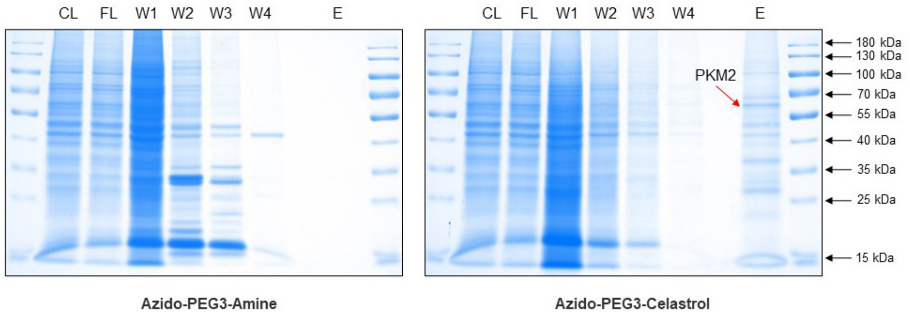

Fig. S1. Coomassie blue detection of celastrol-bound proteins. CL: cell lysates; FL: flow-through fraction; W1-W4: washing fractions; E: elution fraction.

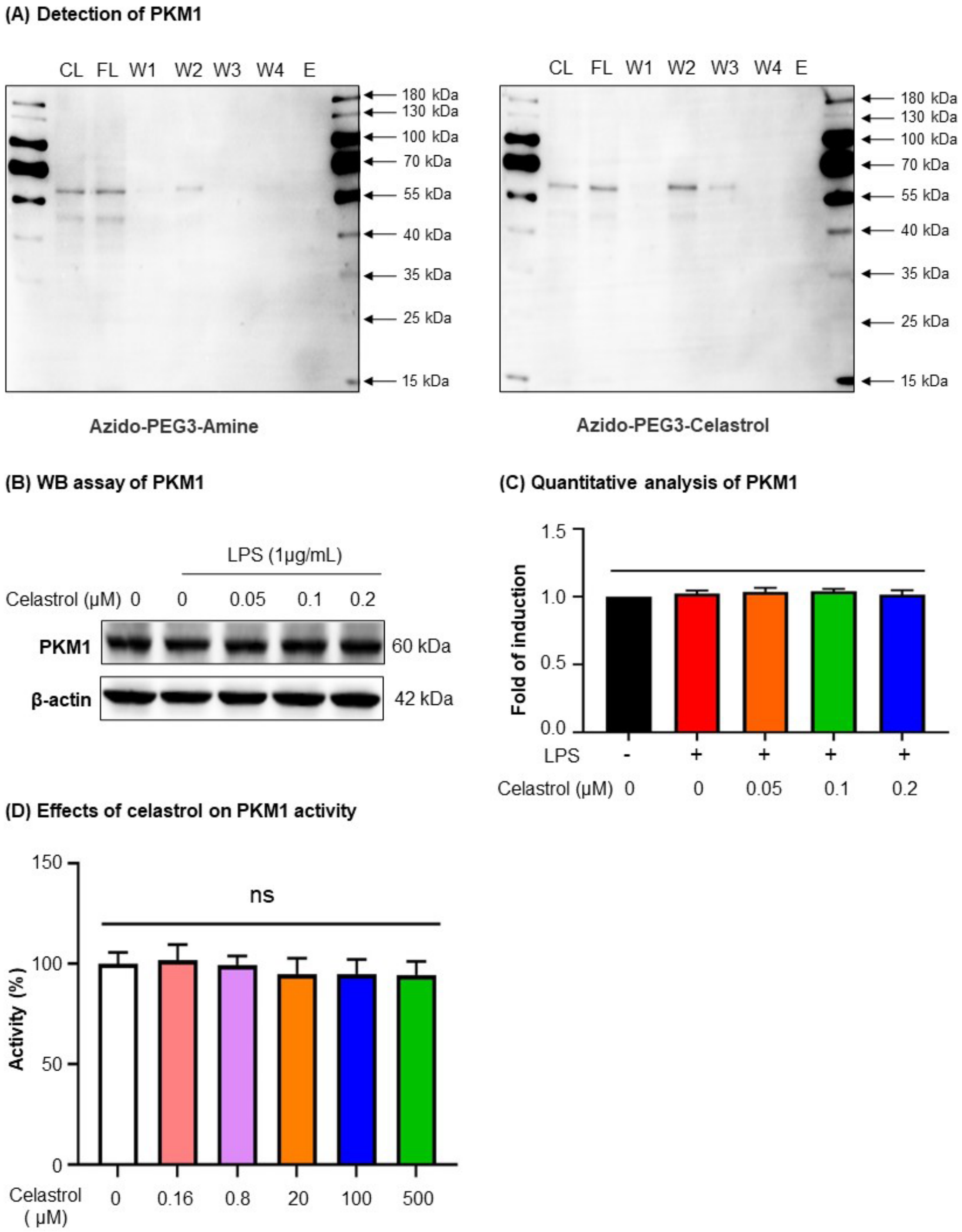

Fig. S2. Western blot analysis of celastrol-bound proteins, effects of celastrol on PKM1 expression and activity. (A) WB analysis of celastrol-bound proteins using PKM1 antibody. CL: cell lysates; FL: flow-through fraction; W1-W4: washing fractions; E: elution fraction. (B) Effects of celastrol on the cellular PKM1 expression. After treated celastrol and LPS as stated in "Methods", RAW264.7 cells were lysed and examined by WB analysis. (C) Quantitative analysis results of PKM1 (n=3). (D) Effects of celastrol on PKM1 activity. After overnight incubation with celastrol, recombinant PKM1 protein was assayed for pyruvate kinase activity.

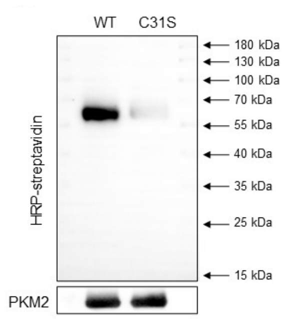

Fig. S3. Detection the binding ability of celastrol with wild type PKM2 and cysteine31 mutated PKM2. Wild type PKM2 (WT) or cysteine31 mutated PKM2 (C31S) were treated with celastrol-PEG3-biotin at 4 °C overnight, respectively. The celastrol-PKM2 conjugate was examined by WB analysis using streptavidin-HRP or anti-PKM2 antibody.

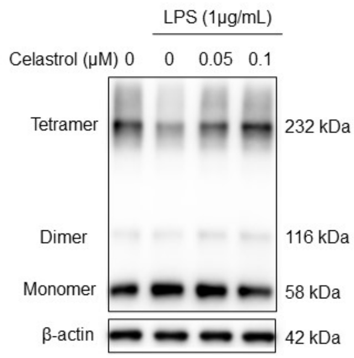

Fig. S4. Effects of celastrol on PKM2 monomer/tetramer. After treatment with celastrol, the RAW264.7 cell lysates were cross-linked with 2.5 mM disuccinimidyl suberate and blotted with anti-PKM2 antibody.
